# What is new in acute myeloid leukemia classification?

**DOI:** 10.1007/s44313-024-00016-8

**Published:** 2024-04-15

**Authors:** Hee Sue Park

**Affiliations:** 1 Department of Laboratory Medicine, Chungbuk National University Hospital, 776, 1 Sunhwan-ro, Seowon-gu, Cheongju, Chungcheongbuk-do 28644 Republic of Korea; 2 Department of Laboratory Medicine, Chungbuk National University College of Medicine, 776, 1 Sunhwan-ro, Seowon-gu, Cheongju, Chungcheongbuk-do 28644 Republic of Korea

**Keywords:** Acute myeloid leukemia, International Consensus Classification, WHO classification

## Abstract

Recently, the International Consensus Classification (ICC) and the 5^th^ edition of the World Health Organization classification (WHO2022) introduced diagnostically similar yet distinct approaches, which has resulted in practical confusion. This review compares these classification systems for acute myeloid leukemia (AML), building up on the revised 4th edition of WHO (WHO2016). Both classifications retain recurrent genetic abnormalities as a primary consideration. However, they differ in terms of blast threshold. The ICC mandates a minimum of 10% blasts in the bone marrow or peripheral blood, whereas the WHO2022 does not specify a blast cut-off. AML with *BCR::ABL1* requires > 20% blast count in both classifications. In WHO2022, AML with *CEBPA* mutation requires > 20% blasts. *TP53* mutation, a new entity is exclusive to ICC, diagnosed with > 20% blasts and variant allele frequency > 10%. AML with myelodysplasia-related changes is defined by cytogenetic or gene mutation-based criteria, not morphological dysplasia. Eight genes were common to both groups: *ASXL1*, *BCOR*, *EZH2*, *SF3B1*, *SRSF2*, *STAG2*, *U2AF1*, and *ZRSR2*. An additional gene, *RUNX1*, was included in the ICC classification. AML cases defined by differentiation (WHO2022) and AML not otherwise specified (ICC) are categorized as lacking specific defining genetic abnormalities, WHO2022 labels this as a myeloid neoplasm post cytotoxic therapy (MN-pCT), described as an appendix after specific diagnosis. Similarly, in ICC, it can be described as “therapy-related”, without a separate AML category.

## Introduction

The World Health Organization (WHO) classification of hematolymphoid tumors has long served as an international diagnostic criterion. However, in 2022, the International Consensus Classification (ICC) and the 5^th^ edition of the WHO classification (WHO2022) offered similar but distinct diagnostic approaches, leading to confusion [1–5]. Since the French-American-British classification in 1976, subsequent updates like WHO2001, WHO2008, and WHO2016, have incorporated new diagnostic criteria that integrate molecular, pathological, and clinical variables into a morphological classification [6–9]. The myeloblast threshold in diagnostic criteria has gradually decreased, with genetic abnormalities emerging as a crucial criterion. The evolution has made personalized management more feasible over time. This review explores the changes from the revised 4th edition of WHO2016 to WHO 2022 and the ICC classification, focusing on acute myeloid leukemia (AML).

## WHO2016: acute myeloid leukemia with recurrent genetic abnormalities

Genetic abnormalities continue to be key diagnostic criteria. The WHO2016 classification, which defined “AML with recurrent genetic abnormalities”, was renamed “AML with defining genetic abnormalities” in WHO2022 [10]. While maintaining the same ICC, additional “other rare recurring translocations” subgroups were created [5]. Both WHO2022 and ICC were broader in scope compared to WHO2016 (Table 1).

**Table 1:** Classification of acute myeloid leukemia with recurrent genetic abnormalities

**WHO2016**		**WHO2022**		**ICC**	
Classification	Blast (%)	Classification	Blast (%)	Classification	Blast (%)
**AML with recurrent genetic abnormalities**		**AML with defining genetic abnormalities**		**AML with recurrent genetic abnormalities**	
APL with *PML::RARA*	.	APL with *PML::RARA* fusion	.	APL with t(15;17)(q24.1;q21.2)/*PML::RARA* APL with other* RARA* rearrangement	≥ 10
AML with t(8;21)(q22;q22.1)/*RUNX1::RUNX1T1*	.	AML with *RUNX1::RUNX1T1* fusion	.	AML with t(8;21)(q22;q22.1)/*RUNX1::RUNX1T1*	≥ 10
AML with inv(16)(p13.1q22) or t(16;16)(p13.1;q22)/*CBFB::MYH11*	.	AML with *CBFB::MYH11* fusion	.	AML with inv(16)(p13.1q22) or t(16;16)(p13.1;q22)/*CBFB::MYH11*	≥ 10
AML with t(9;11)(p21.3;q23.3);*KMT2A::MLLT3*	**≥ 20**	AML with *KMT2A* rearrangement	.	AML with t(9;11)(p21.3;q23.3)/*MLLT3::KMT2A* AML with other *KMT2A* rearrangement	≥ 10
AML with t(6;9)(p23;q34.1);*DEK::NUP14*	**≥ 20**	AML with *DEK::NUP214* fusion	.	AML with t(6;9)(p23;q34.1)/*DEK::NUP14*	≥ 10
AML with inv(3)(q21.3q26.2) or t(3;3)(q21.3;q26.2);*GATA2::MECOM*	**≥ 20**	AML with *MECOM* rearrangement	.	AML with inv(3)(q21.3q26.2) or t(3;3)(q21.3;q26.2)/*GATA2*;*MECOM*(*EVI1*) AML with other *MECOM* rearrangement	≥ 10
AML with t(9;22)(q34.1;q11.2)/*BCR::ABL1*	**≥ 20**	AML with *BCR::ABL1* fusion	**≥ 20**	AML with t(9;22)(22)(q34.1;q11.2)/*BCR::ABL1*	**≥ 20**
AML with t(1;22)(p13.3;q13.1);*RBM15::MLK1*	**≥ 20**	AML with *RBM15::MRTFA* fusion	.		≥ 10
AML with mutated *NPM1*	.	AML with *NPM1* mutation		AML with mutated *NPM1*	≥ 10
AML with biallelic mutation of *CEBPA*	.	AML with *CEBPA* mutation	**≥ 20**	AML with mutated bZIP *CEBPA*	≥ 10
(provisional) AML with mutated *RUNX1*	**≥ 20**	AML with *NUP98* rearrangement	.	AML with *TP53*	**≥ 20**
		AML myeloid leukemia, myelodysplasia-related	**≥ 20**	**AML with other rare recurring translocations**	≥ 10
		AML with other defined genetic alteration	**≥ 20**	AML with t(1;3)(p36.3;q21.3)/*PRDM16::RPN1*	
				AML with t(3;5)(q25.3)(q25.3;q35.1)/*NPM1::MLF1*	
				AML with t(8;16)(p11.2;p13.3)/*KAT6A::CREBBP*	
				AML with t(1;22)(p13.3;q13.1)/*RBM15::MRTF1*	
				AML with t(5;11)(q35.2;p15.4)/*NUP98:NSD1*	
				AML with t(11;12)(p15.4;p13.3)/*NUP98::KMD5A*	
				AML with *NUP98* and other partners	
				AML with t(7;12)(q36.3;p13.2)/*ETV6::MNX*	
				AML with t(10;11)(p12.3);q14.2)/*PICALM::MLLT10*	
				AML with t(16;21)(p11.2;q22.2)/*FUS::ERG*	
				AML with t(16;21)(q24.3;q22.1)/*RUNX1::CBFA2T3*	
				AML with inv(16)(p13.3;q24.3)/*CBFA2T3::GLIS2*	

*Abbreviations*: *AML* Acute myeloid leukemia, *APL* Acute promyelocytic leukemia

The key change in WHO2022 is the exclusion of the myeloblast percentage threshold for diagnosis when specific genetic abnormalities are present. Unlike WHO2016, where the myeloblast count was not a significant factor in diagnosing certain AML subtypes, such as AML with t(8;21)(q22;q22.1), AML with inv(16)(p13.1q22) or t(16;16)(p13.1;q22), and acute promyelocytic leukemia (APL) with *PML-RARA*, WHO2022 now applies myeloblast count criteria to additional genetic abnormalities such as t(9;11)(p21.3;q23.2), t(6;9)(p23;q34.1), inv(3)(q21.3q26.2), t(3;3)(q21.3;q26.2) or t(1;22)(p13.3;q13.1), while excluding AML with *BCR::ABL1* fusion and *CEBPA* mutation. A novel structure for “AML with other defined genetic alterations” was introduced, including new and/or uncommon AML subtypes that may be included in future editions. The ICC further categorized subgroups into “AML with recurrent genetic abnormalities” and “other rare recurring translocations”. Notable differences include 1) the incorporation of additional *RARA*, *KMT2A*, and *MECOME* rearrangements and 2) a requirement for blast count exceeding 10% for diagnosis, except in cases of t(9;22)(22)(q34.1;q11.2)/*BCR::ABL1* and *TP53* mutations, which require a blast count exceeding 20%.

In WHO2016 classification, AML with mutated *NPM1*, AML with biallelic mutations in *CEBPA*, and AML with mutated *RUNX1* were classified as AML with genetic mutations. AML with mutated *NPM1* and *CEBPA* remained classified in both the WHO2022 and ICC classifications. However, AML with mutated *RUNX1* was excluded from the provisional diagnosis due to its limited clinical significance.

### AML with mutated *NPM1*

*NPM1*-mutated AML has been recognized as a distinct entity since 2008. Morphologically, blasts exhibit monocytic differentiation, and this subtype is frequently observed in young patients with a high prevalence of the normal karyotype [11–13]. There is a discrepancy in the blast threshold for diagnosis between the ICC and WHO2022. ICC requires a blast count ≥ 10%, whereas WHO2022 does not specify a blast number cutoff. Although an increase in blasts exists in most AML cases with mutated *NPM1*, if the blast count is < 10%, the diagnosis is changed to “AML with *NPM1”* in WHO2022 and “*NPM1*-mutated myelodysplastic syndrome (MDS)” in ICC. *NPM1* mutations are also detected in MDS and MDS/MPN [14], occurring in approximately 2% of MDS, cases with excess blasts [15], leading to potential confusion in clinical management and treatment decisions.

### Especially concerning AML with biallelic mutation of *CEBPA*

In WHO2022, this category is termed “AML with *CEBPA* mutation”, encompassing biallelic (bi*CEBPA*) and single mutations in the basic leucine zipper region (smbZIP-*CEBPA*) [10]. Conversely, ICC designates the diagnosis as “AML with mutated bZIP *CEBPA*”, emphasizing the bZIP domain mutation irrespective of its mono or biallelic nature. This conclusion is supported by recent studies demonstrating that bZIP domain mutations are linked to favorable clinical outcomes [4, 16]. The blast count diagnostic criteria in ICC, consistent with other entities, is ≥ 10%. In contrast, WHO2022 suggests a blast count of ≥ 20%. Common morphological features often indicate AML with maturation (FAB M2) or AML without maturation (FAB M1) [17]. However, distinctive morphological features are lacking and occur at a frequency of 7–16% in adults and 4.5–15% in pediatric patients [4].

### AML with *TP53* mutation

Notably*, TP53* was not included in the WHO2022 AML with defined genetic abnormalities. Instead, a biallelic *TP53* alteration subtype is recognized in MDS, which is considered equivalent to AML. The diagnostic criteria for *TP53* alterations in ICC require a blast count ≥ 20%, a higher threshold than in other entities, in conjunction with a variant allele frequency ≥ 10%. Therefore, when the blast count is < 20% in peripheral blood and bone marrow, MDS is characterized by both classifications. In WHO2022, “MDS with biallelic *TP53* inactivation (MDS-bi*TP53*)” is defined for cases with < 20% blasts, whereas ICC delineates “MDS mutated *TP53*” according to blast count differences. In addition, in cases of monoallelic loss, there was no significant clinical difference compared to the wild type [18]. Therefore, both classifications focus on biallelic loss.

### AML with *NUP98* rearrangement

This category is a newly introduced as “AML with *NUP98* rearrangement” in WHO2022 and as “AML with t(5;11)(q35.2;p15.4)/*NUP98*::*NSD1* and with t(11;12)(p15.4;p13.3)/*NUP98*:*KMD5A* and *NUP98* and other partners” in ICC. *NUP98* exhibits multiple fusion partners and, although infrequent, is associated with a poor prognosis [19]. The blast count requirement was maintained as a minimum for both classifications.

## WHO2016: AML with myelodysplasia-related changes

In WHO2022, this category was named “AML with myelodysplasia-related (AML-MR)”, and ICC classified it as “AML with myelodysplasia-related gene mutation” and “AML with myelodysplasia- related cytogenetic abnormalities”. It was incorporated from an independent category into “AML with defining genetic abnormalities” in WHO2022. Both classification systems exclude morphology-based diagnostic criteria and emphasize molecular abnormalities. Some existing cytogenetic criteria have been updated, and gene mutations have been added. The myeloblast threshold requires ≥ 20% in both peripheral blood or bone marrow for this category.

### AML with myelodysplasia-related cytogenetic abnormalities

Although there were no significant differences from the previous WHO2016, some distinctions were observed between the two classification systems (Table 2). Complex karyotype (≥ 3 abnormalities) and chromosomal aberrations on chromosomes 5, 7, 12, 17, and X were common in both systems. In the ICC, del(11q) was excluded, and +8 and del(20q) were added. Additionally, balanced abnormalities in WHO2016 were moved to “AML with other rare recurring translocations”.

**Table 2:** Classification of acute myeloid leukemia with myelodysplasia-related changes

**WHO2016**	**WHO2022**	**ICC**
**Complex karyotype (≥ 3 abnormalities)**	**Cytogenetic abnormalities**	**Cytogenetic abnormalities**
**Unbalanced abnormalities**	Complex karyotype (≥ 3 abnormalities)	Complex karyotype (≥ 3 abnormalities)
del(5q) or t(5q)	5q deletion or loss of 5q	del(5q)/t(5q)/add(5q)
Loss of chromosome 7 or del(7q)	monosomy 7, 7q deletion, or loss of 7q	-7/del(7q)
Loss of chromosome 13 or del(13q)	11q deletion	+8
del(11q)	12p deletion or loss of 12p	del(12p)/t(12p)/add(11p)
del(12p) or t(12p)	Monosomy 13 or 13q deletion	i(17q), -17/add(17p) or del(17p)
isochromosome 17q or t(17p)	17p deletion or loss of 17p, isochromosome 17q	del(20q)
idic(X)(q13)	idic(X)(q13)	idci(X)(q13)
**Balanced abnormalities**	**Defining somatic mutations**	**Gene mutations**
t(11;16)(q23.3;p13.3)	*ASXL1*	*ASXL1*
t(3;21)(q26.2;q22.1)	*BCOR*	*BCOR*
t(1;3)(p36.3;q21.2)	*EZH2*	*EZH2*
t(2;11)(p21;p23.3)	*SF3B1*	*RUNX1*
t(5;12)(q32;p13.2)	*SRSF2*	*SF3B1*
t(5;7)(q32;q11.2)	*STAG2*	*SRSF2*
t(5;17)(q32;p13.2)	*U2AF1*	*STAG2*
t(5;10)(q32;q21)	*ZRSR2*	*U2AF1*
t(3;5)(q25.3;q35.1)		*ZRSR2*

### AML with myelodysplasia-related gene mutations

Eight genes were common to both groups: *ASXL1*, *BCOR*, *EZH2*, *SF3B1*, *SRSF2*, *STAG2*, *U2AF1*, and *ZRSR2*. An additional gene, *RUNX1*, was included in ICC. Minimum variant allele frequencies are not required for these genes. They are associated with an adverse prognosis [20, 21].

## WHO2016: therapy-related myeloid neoplasm

In WHO2022, a category named “Myeloid neoplasms post cytotoxic therapy (MN-pCT)” was introduced, encompassing AML, MDS, and MDS/MPN that develop after cytotoxic therapy inducing DNA damage [10]. Cytotoxic therapies, such as PARP1 inhibitors and methotrexate, were excluded. It is recommended to append “post cytotoxic therapy” after the specific diagnosis. Similarly, the ICC no longer recognized it as a distinct entity of AML.

## WHO2016: acute myeloid leukemia, not otherwise specified

This group lacked genetic abnormalities and was classified based on morphology. Although this subtype has limited prognostic significance, it offers a practical paradigm [5, 10]. Both WHO and ICC maintain diagnostic criteria of ≥ 20% myeloblasts. Additionally, this category includes cases with overlapping phenotypic markers of the two lineages, such as mixed phenotype acute leukemia (MPAL) and early T-precursor lymphoblastic leukemia/lymphoma (ETT-ALL). Until recently, the genomics of MPAL has been predominantly associated with *KMT2A* rearrangement. However, recent findings have highlighted the involvement of the RAS pathway in B/M MPAL, the JAK/STAT pathway in T/M MPAL, *ZEB2-BCL11B*, *NUP214-ABL1*, and *ETV6* in T/myeloid cells. These discoveries suggest the potential for future addition of new entities [10, 22].

## European LeukemiaNet (ELN) risk stratification 2022

Aligned with the updated AML classification system that emphasizes genetic mutations, the ELN has released 2022 risk stratification guidelines based on the ICC classification (Table 3) [23, 24]. Key changes included: 1) retention of recurrent genetic abnormalities and the addition of new genetic mutations. Eight genes were included in the adverse risk category and designated as AML with myelodysplasia-related gene mutations. 2) The prognostic division based on the allelic ratio of *FLT3-ITD* in cases of AML coexisting with mutated *NPM1* and *FLT3-ITD* was eliminated in ELN2017. This is due to the lack of standardization in the method for measuring the *FLT*-*ITD* allelic ratio. 3) Additionally, *NPM1* mutated AML with additional adverse-risk cytogenetic abnormalities was classified as an adverse risk. 4) Mutations in the basic leucine zipper region of *CEBPA* that affect in-frame confer a favorable prognosis, regardless of whether monoallelic or biallelic mutations. 5) Additional cytogenetic abnormalities such as t(3q26.2;v)/*MECOME*-rearranged and t(8;16)(p11.2;p13.3)/*KAT6A*::*CREBBP* fusion, are now included in the adverse risk group [25, 26]. 6) Hyperdiploidy with multiple trisomies is not considered a complex karyotype.

**Table 3:** Comparison of 2017 and 2022 European LeukmiaNet-acute myeloid leukemia risk classification

**Risk category**	**2017 ELN**	**2022 ELN**
**Favorable**	t(8;21)(q22;q22.1);*RUNX1-RUNX1T1*	t(8;21)(q22;q22.1)/*RUNX1::RUNX1T1*
	inv(16)(p13.1q22) or t(16;16)(p13.1;q22);*CBFB-MYH11*	inv(16)(p13.1q22) or t(16;16)(p13.1;q22)/*CBFB::MYH11*
	Mutated *NPM1* without *FLT3*-ITD or with *FLT3*-ITD^low*^	Mutated *NPM1* without *FLT3*-ITD
	Biallelic mutated *CEBPA*	bZIP in-frame mutated *CEBPA*
**Intermediate**	Mutated *NPM1* with *FLT3*-ITD^high^	Mutated *NPM1* with *FLT3*-ITD
	Wild-type *NPM1* without *FLT3*-ITD or with FLT3-ITD^low^ (without adverse-risk genetic lesions)	Wild-type *NPM1* with *FLT3*-ITD (without adverse-risk genetic lesions)
	t(9;11)(p21.3;q23.3)/*MLLT3::KMT2A*	t(9;11)(p21.3;q23.3)/*MLLT3::KMT2A*
	Cytogenetic abnormalities not classified as favorable or adverse	Cytogenetic and/or molecular abnormalities not classified as favorable or adverse
**Adverse**	t(6;9)(p23.3;q34.10);*DEK-NUP214*	t(6;9)(p23.3;q34.10)/*DEK::NUP214*
	t(v;11q23.3);*KMT2A*-rearranged	t(v;11q23.3)/*KMT2A*-rearranged
	t(9;22)(q34.1;q11.2);*BCR-ABL1*	t(9;22)(q34.1;q11.2)/*BCR::ABL1*
		t(8;16)(p11.2;p13.3)/*KAT6A::CREBBP*
	inv(3)(q21.3q26.2) or t(3;3)(q21.3;q26.2);*GATA2, MECOM(EVI1)*	inv(3)(q21.3q26.2) or t(3;3)(q21.3;q26.2)/*GATA2, MECOM(EVI1)*
		t(3q26.2;v)/*MECOME*(*EVI1*)-rearranged
	-5 or del(5q); -7; -17/abn(17p)	-5 or del(5q); -7; -17/abn(17p)
	Complex karyotype, monosomal karyotype	Complex karyotype, monosomal karyotype
	Mutated *RUNX1*, *ASXL1*	Mutated *ASXL1*, *BCOR*, *EZH2*, *RUNX1*, *SF3B1*, *SRSF2*, *STAG2*, *U2AF1*, and/or *ZRSR2*
	Mutated *TP53*	Mutated *TP53*

^*^FLT3: *low* Low allelic ratio (< 0.5), *high* High allelic ratio(≥ 0.5)

## Conclusion

In recent years, studies on the genetic spectrum of AML have increased, expanding the treatment possibilities [27]. Various gene-targeted therapies, such as FLT3 inhibitors, are being introduced in chemotherapy regimens and are undergoing continuous clinical trials [28, 29]. While both the WHO2022 and ICC classification systems are based on these findings, these new classifications have added complexity for researchers and physicians. Differences in terminology and the introduction of updated/new diagnostic entities can cause confusion in the field, affecting diagnosis, management, clinical outcome assessment, and clinical trials [2, 3]. Additionally, the diagnosis and risk stratification of AML require various molecular tests, which depend on the adequate economic and diagnostic capacity for their execution. Molecular tests, taking more than two weeks for a formal report, may result in a delayed diagnosis compared to traditional morphological diagnoses, potentially delaying treatment. The new classification system integrates morphological, immunophenotypic, molecular, and cytogenetic information, facilitating the adoption of precision medicine. Consequently, treatment decisions should be based on comprehensive laboratory tests, medical histories, and clinical information.

## Data Availability

No datasets were generated or analysed during the current study.
